# Infancy of peracetic acid activation by iron, a new Fenton-based process: A review

**DOI:** 10.1016/j.heliyon.2024.e27036

**Published:** 2024-02-24

**Authors:** Iván Sciscenko, Davide Vione, Marco Minella

**Affiliations:** a Departamento de Ingeniería Textil y Papelera, Universitat Politècnica de València, plaza Ferrándiz y Carbonell S/N, 03801, Alcoy, Spain; b Department of Chemistry, University of Turin, via Pietro Giuria 5, 10125, Turin, Italy

**Keywords:** Advanced oxidation processes, Emerging pollutants, Ferryl, Peracids, Reactive oxygen species, Water treatment

## Abstract

The exacerbated global water scarcity and stricter water directives are leading to an increment in the recycled water use, requiring the development of new cost-effective advanced water treatments to provide safe water to the population. In this sense, peracetic acid (PAA, CH_3_C(O)OOH) is an environmentally friendly disinfectant with the potential to challenge the dominance of chlorine in large wastewater treatment plants in the near future. PAA can be used as an alternative oxidant to H_2_O_2_ to carry out the Fenton reaction, and it has recently been proven as more effective than H_2_O_2_ towards emerging pollutants degradation at circumneutral pH values and in the presence of anions. PAA activation by homogeneous and heterogeneous iron-based materials generates - besides HO^•^ and FeO^2+^ - more selective CH_3_C(O)O^•^ and CH_3_C(O)OO^•^ radicals, slightly scavenged by typical HO^•^ quenchers (e.g., bicarbonates), which extends PAA use to complex water matrices. This is reflected in an exponential progress of iron-PAA publications during the last few years. Although some reviews of PAA general properties and uses in water treatment were recently published, there is no account on the research and environmental applications of PAA activation by Fe-based materials, in spite of its gratifying progress. In view of these statements, here we provide a holistic review of the types of iron-based PAA activation systems and analyse the diverse iron compounds employed to date (e.g., ferrous and ferric salts, ferrate(VI), spinel ferrites), the use of external ferric reducing/chelating agents (e.g., picolinic acid, l-cysteine, boron) and of UV–visible irradiation systems, analysing the mechanisms involved in each case. Comparison of PAA activation by iron vs. other transition metals (particularly cobalt) is also discussed. This work aims at providing a thorough understanding of the Fe/PAA-based processes, facilitating useful insights into its advantages and limitations, overlooked issues, and prospects, leading to its popularisation and know-how increment.

## Highlights

• Uses of peracetic acid-based Fenton-like processes are reviewed.
• Types of employed iron catalysts are compared.
• Mechanistic aspects of Fe/peracetic acid are reviewed and discussed.
• Discussion of bottlenecks and prospects related to Fe/peracetic acid is given.

## 1 Introduction

The use of reclaimed water (treated wastewater) for irrigation purposes is one of the best established methods to tackle down water scarcity, both for arid/semi-arid regions and for traditionally water-rich zones that are currently facing long periods of drought, as a consequence of anthropogenic climate change. However, treated wastewater reuse has several drawbacks, including crops contamination with pathogenic microorganisms (in countries where wastewater disinfection is not mandatory or insufficiently implemented) and exposure to the so-called contaminants of emerging concern (CECs), such as perfluoroalkyl substances, plasticisers, pesticides, and pharmaceuticals [1]. These issues also apply to tap/drinking water: concerns about its quality have primarily been limited to microbial content, concentration of disinfection by-products (DBP, such as trihalomethanes), and major ions (e.g., nitrates or heavy metals). However, lack of thorough regulatory frameworks for CECs in wastewater and drinking water, together with the relatively low CEC removal efficiency of traditional technologies, have led to worldwide occurrence of these compounds in water environments [2,3]. In this regard, the European Union have recently promulgated a regulation for reclaimed wastewater quality standards, which has entered in force at the end of June 2023 [4], where some CECs will be regulated. Similar regulation has been enforced in January 2021 for drinking water [5], and a revision of the EU directive for urban waste water depuration and management is expected soon.

Advanced Oxidation Processes (AOPs) rely on the generation of very powerful oxidant species (often radicals like the hydroxyl radical –HO^•^– and other Reactive Oxygen Species – ROS) and can be coupled to urban-wastewater and drinking-water treatment plants (UWTP and DWTP, respectively) to simultaneously abate CECs and microorganisms (including antibiotic resistant bacteria –ARB– and genes –ARGs) [6,7]. AOPs use has also been extended to other niche applications, such as industrial wastewater [8], air [9], and soil remediation [10]. Among AOPs, the Fenton reaction consists in the decomposition of hydrogen peroxide (H_2_O_2_) catalysed by iron ions. The proposed mechanisms usually involve the elementary steps R1-R12 with formation of HO^•^, HO_2_^•^, and high-valent iron (e.g., FeO^2+^ and FeOFe^5+^) as reactive species able to oxidize CECs [11,12]. Compared to HO^•^ (E° = 2.80 V vs. NHE and bimolecular rate constants with CECs of ca. 1 × 10^9^ M^−1^ s^−1^), high-valent iron species are weaker oxidants (E° ≥ 1.2 V vs. NHE) and more selective (k ≈ (0.001–1) × 10^5^ M^−1^ s^−1^), thus, with higher lifetimes [13,14].

R1 Fe^2+^ + H_2_O_2_ + H^+^ → Fe^3+^ + HO^•^ + H_2_O ; k = 63–76 M^−1^ s^−1^

R2 Fe^3+^ + H_2_O_2_ → Fe^2+^ + HO_2_^•^ + H^+^ ; k = 0.001–0.01 M^−1^ s^−1^

R3 HO^•^ + H_2_O_2_→ HO_2_^•^ + H_2_O

R4 HO^•^ + Fe^2+^ → Fe^3+^ + OH^−^

R5 Fe^3+^ + HO_2_^•^ → Fe^2+^ + O_2_ + H^+^

R6 Fe^2+^ + HO_2_^•^ + H^+^ → Fe^3+^ + H_2_O_2_

R7 2 HO_2_^•^ → H_2_O_2_ + O_2_

R8 Fe^2+^ + H_2_O_2_ → FeO^2+^ + H_2_O

R9 FeO^2+^ + H_2_O_2_ → Fe^2+^ + H_2_O + O_2_

R10 FeO^2+^ + Fe^2+^ + H_2_O → 2 Fe^3+^ + 2 HO^−^

R11 FeO^2+^ + Fe^3+^ → FeOFe^5+^

R12 FeOFe^5+^ + H_2_O_2_ → Fe^2+^ + Fe^3+^ + O_2_ + H_2_O

The review by Pignatello et al. (2006) is a milestone in the revision of the fundamentals behind the aforementioned reaction, which is also enhanced in presence of light (photo-Fenton), electrical current (electro-Fenton), and ultrasound waves (sono-Fenton) [12]. The two major inconveniencies of every Fenton-based process are, the precipitation of Fe(III) at pH ≥ 4 (which eventually hinders the reaction at circumneutral pH) and the slow Fe(II) regeneration by reaction R2. To overcome these disadvantages, several strategies have been studied, such as replacing iron salts by their oxides or zerovalent iron, ZVI (heterogeneous Fenton), or employing Fe(III) reducing agents (e.g., boron, hydroxylamine, cysteine …) and ligands for Fe(II, III) (e.g., nitrilotriacetic acid - NTA - or ethylenediamine-N,N-disuccinic acid - EDDS) to accelerate Fe(II) regeneration and keep iron ions dissolved, respectively [15,16].

Among the studied Fenton variants, the use of alternative oxidants to H_2_O_2_ has recently received increasing interest. Examples are persulfate (PDS), peroxymonosulfate (PMS), or sulfite (SO_3_^2−^). Fe/PDS and Fe/PMS produce the reactive sulfate radical (SO_4_^•−^, E° = 2.5–3.1 V vs NHE; k = (0.01–1) × 10^9^ M^−1^ s^−1^) [17], whereas Fe/SO_3_^2−^ is interesting because of its low cost and wide ROS generation (e.g., HO^•^, SO_4_^•−^, SO_3_^•−^, and SO_5_^•−^) [18,19]. Lately, peracetic acid (CH_3_C(O)OOH, also known as peroxyacetic acid, hereinafter PAA) has been proposed as well.

PAA is a strong oxidant formed from acetic acid (HAc) and H_2_O_2_ under acidic conditions [20], and it is a very promising substitute for chlorine in wastewater treatment due to its comparable disinfection power, negligible hazardous DBP formation, and avoidance of the need to neutralize excess oxidant in the effluent [21,22]. An extensive review of the most used PAA-AOP processes has been provided by the group who most studied them [23]. Compared to H_2_O_2_, PAA has lower O–O bond energies (213 and 159 kJ mol^−1^, respectively) with easier homolysis by diverse activation methods (e.g., UV or presence of transition metals). In fact, Fe/PAA reactions (equivalent to R1 and R2) are several orders of magnitude faster than Fe/H_2_O_2_, with higher CEC removal rates at neutral pH values [24]. Moreover, further reactive radicals are formed, such as CH_3_C(O)O^•^ and CH_3_C(O)OO^•^, which are more selective than HO^•^ and are not scavenged significantly by common anions like bicarbonates [25]. Therefore, they are more suitable for complex water matrices. However, PAA-AOPs also rise the final total organic carbon (TOC) due to residual PAA and the resulting intermediates (e.g., formaldehyde or acetic acid) [26,27]. Although acetic acid is biodegradable, TOC is a regulated parameter in most water-quality laws, thus this issue should be borne in mind when employing peracids for water treatment. Furthermore, the measurement of TOC as an indicator of treatment progress (i.e., mineralisation) and performance is hardly feasible when using PAA as reagent.

Published works dealing with PAA-Fenton have risen to 129 between 2019 and 2022, approximately two times more than in the previous 40 years (based on Scopus) placing PAA-Fenton in an infant stage. Although five detailed reviews have been very recently published, covering PAA-based AOPs [23,[28], [29], [30], [31]], to the best of our knowledge, there is no specific account on PAA activation by Fe-based materials. Therefore, this paper presents a holistic review on the iron-based PAA activation systems reported so far, the employed iron compounds (salts, ZVI, oxides, and minerals), the use of external ferric reducing/chelating agents, the enhancement with light, the involved mechanisms, and CEC bimolecular rate constants. Comparisons of PAA activation by Fe and other transition metals are also provided, highlighting advantages, limitations, and lack of information where applicable. The final aim is also to propose, on the basis of an overall literature analysis, aspects of interest for future research works aimed at a more efficient and fast development of knowledge about this process.

## 2 Peracetic acid (CH_3_C(O)OOH)

### 2.1 Brief overview

PAA is an oxidant used in disinfection, sterilisation, bleaching, and chemical synthesis. Water treatment represents the 17% of PAA global market share, right after food industry (32%) and the healthcare sector (24%) [29]. PAA is produced from H_2_O_2_ and acetic acid (HAc) under acidic catalysis, usually with H_2_SO_4_ 0.5–1 mol L^−1^ (R13) [20,32]. It is typically commercialised as 5–35% active content solutions, with PAA/H_2_O_2_ molar ratios from 0.1 to 3 and trace amounts of stabilisers (>15% PAA solutions are unstable, R14) [33]. Noteworthy, because of issues with H_2_SO_4_ (and/or HAc), which corrodes the equipments, the production of pure PAA solutions can be obtained through vacuum distillation column processes, which is required for some industries such as the fine chemistry ones [20].

PAA was introduced as a promising disinfectant for UWTP and DWTP in the early 1980s, and since then it has been proposed for the disinfection of, among others: secondary wastewater effluents [34,35], UWTP sludge [36], drinking water [37,38], recirculating aquaculture systems (RAS) [39,40], and ballast water [41,42]. Although PAA is more environmentally benign than chlorine or ozone, several recent articles have reported overlooked formation of DBPs (CHCl_3_, CHBr_3_) when PAA was added to very saline groundwater (containing ca. 3 g L^−1^ Cl^−^ and 6 mg L^−1^ Br^−^ [43]), or trichloronitromethane formation when NO_2_^−^ (0.23–920 mg L^−1^) is present [44]. Therefore, plausible formation of DBPs when using PAA in highly-saline water matrices must be evaluated.

The reported PAA standard reduction potential ranges from 1.06 [32] to 1.96 V vs NHE [23]. This wide interval is plausibly related to the pH-dependence of the PAA equilibrium with H_2_O_2_ and HAc (R13; PAA formation is favoured at acidic pH while its decomposition at alkaline one) leading to uncertainties on its measurement. Recently, a thorough work has reported the thermodynamic properties of PAA, with E = 1.385 V vs. NHE at pH = 7.25, T = 25°C and P = 1 bar (R15), higher at pH = 0 (E° = 1.748 V vs NHE), and lower at pH = 14 (E° = 1.005 V vs NHE for PAA^−^) [45]. Further works evaluating thermodynamic properties of PAA are, therefore, required to analyse the accuracy of these values.R13
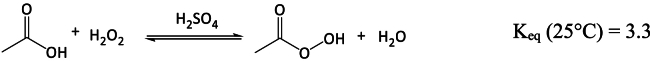
R14
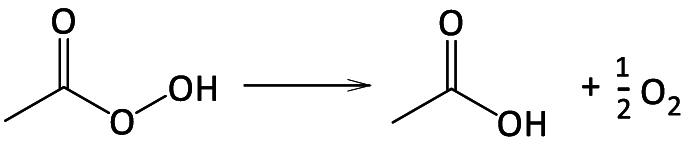
R15
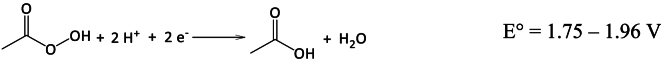
R16
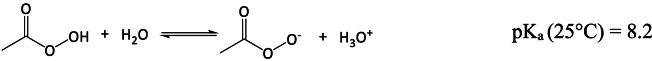


### 2.2 Comparison with other oxidants

Table 1 shows a summary of the properties of some common oxidants. PAA has a reduction potential higher than chlorine (E° = 1.48 V vs NHE), and comparable to that of H_2_O_2_ (E° = 1.78 V vs NHE), PDS (E° = 2.1 V vs NHE), PMS (E° = 1.8 V vs NHE), or O_3_ (E° = 2.08 V vs NHE). Noteworthy, regarding costs (per mol of reagent), PAA (0.55 € mol^−1^) is considerably more expensive than conventional O_3_ (0.08 € mol^−1^), H_2_O_2_ (0.04 € mol^−1^), or HClO (0.07 € mol^−1^). PAA cost (0.8–1 € L^−1^ of 12% PAA, for which one must bear in mind it also contains 20% H_2_O_2_ with further oxidising activity) is also related to the currently low PAA production at the global level. It is estimated that PAA prices should decrease significantly as the worldwide production capacity increases [28,29]. In fact, the global market of PAA was worth $650 million in 2017 and is expected to grow up to $1.3 billion by 2026 [21]. Another drawback is that the use of PAA as diluted solution entails higher costs for logistics (transport and specific storage conditions) compared to “pure” solid oxidants (e.g., calcium hypochlorite, potassium persulfate or sodium percarbonate). However, this issue can be solved by production on-site.

**Table 1:** Summary of properties of diverse oxidants employed in AOPs. * pKa not reported for HS_2_O_8_^−^, the one of H_2_S_2_O_8_ is −3.5; ** never determined for metaperiodic acid (HIO_4_) (reported pKa values for orthoperiodic acid (H_5_IO_6_) = 1.64, 8.36 and 12.2); ***quantum yields not reported, but the IO_4_^−^ photolytic rate constant k = (0.1–1) × 10^−3^ s^−1^.

Oxidant	Standard redox potential vs. NHE (E°, V)	pKa (25 °C)	O–O dissociation energy (kJ mol^−1^)	Photolysis (λ = 254 nm) quantum yield (mol Einstein^−1^)	Required concentration (mM) for *E. coli* 6 LRV in t = 30 min under UV irradiation	Significant formation of hazardous by-products?	Large-scale cost	References
Peracetic acid (PAA, CH_3_C(O)OOH)	1.75–1.96	8.2	159	0.9–1.2	0.03	No	0.8–1 € L^−1^ ≈ 0.55 € mol^−1^ (as 12% PAA solution)	[29,45,54]
Hydrogen peroxide (H_2_O_2_)	1.78	11.6	213	1.0	0.3	No	0.4 € L^−1^ ≈ 0.04 € mol^−1^ (as 35% H_2_O_2_ solution) 0.1 € kg^−1^ ≈ 0.01 € mol^−1^ (as Na_2_CO_3_·H_2_O_2_)	[27,157]
Persulfate (PDS, S_2_O_8_^2−^)	2.1	*	92	0.5	<0.5	No	0.7 € kg^−1^ ≈ 0.2 € mol^−1^ (as K_2_S_2_O_8_)	[27,158,159]
Peroxymonosulfate (PMS, SO_5_^2−^)	1.82	9.4	377	0.5	0.005	No	2 € kg^−1^ ≈ 0.6 € mol^−1^ (as Oxone)	[27,160]
Hypochlorous acid (HClO)	1.49	7.49	/	1.4	<0.04	Yes	0.1 € kg^−1^ ≈ 0.07 € mol^−1^ (as NaClO or Ca(ClO)_2_)	[27,46,161,162]
Ozone (O_3_)	2.07	/	109	2.0	<0.03	No	1.7 € kg^−1^ ≈ 0.08 € mol^−1^ (calculated based on O_2_ cost and electricity)	[157,[163], [164], [165]]
Periodate (IO_4_^−^)	1.60	**	/	***	<0.02	Yes	4 € kg^−1^ ≈ 0.9 € mol^−1^ (as KIO_4_)	[48,49,166,167]

When employing UV/chlorine (characterised by high photolysis quantum yield [46]), formation of trihalomethanes and adsorbable organic halides (sometimes at higher concentration than in the dark) is widely reported, whereas with UV/PAA the most toxic DBP is formaldehyde that is released at concentrations far below drinking water guidelines [27]. Ozone has also higher photolysis quantum yields than PAA, but might also yield different hazardous by-products if bromide levels are considerable [47]. UV/IO_4_^−^ is also proposed as an outstanding AOP, much more efficient than UV/PAA or UV/PDS, where HO^•^, IO_3_^•^, IO_4_^•^, and O(^3^P) are formed [[48], [49], [50]]. However, periodate salts are very expensive (≈4 € kg^−1^ = 0.9 € mol^−1^), and formation of likely genotoxic and cytotoxic iodinated by-products is frequently reported [51,52]. Therefore, the PAA safest competitors in irradiated systems are H_2_O_2_ (only because of the reagent cost, as commercial PAA contains H_2_O_2_), PMS, and PDS, all of them having lower 254-nm photolysis quantum yields than PAA (Table 1). Moreover, PAA-AOPs are less sensitive to anions than H_2_O_2_-, PMS-, or PDS-based processes (see section 2.3).

Another emerging disinfectant, analogous to PAA, is performic acid (PFA). Although PFA is considered to be more cost-effective than PAA (estimating 0.01 € to disinfect 1 m^3^ of secondary effluent with PFA and 0.02–0.06 € m^−3^ with PAA) and the O–O bond dissociation energy for both peracids should be similar (calculations suggest 159 kJ mol^−1^) [29], there is no information on PFA use in AOPs. Therefore, further research is needed on plausible PFA activation methods.

### 2.3 Effect of operational parameters on advanced oxidation processes employing peracetic acid

Operational conditions (concentration of catalyst or PAA, respectively, and pH) usually have analogous influence in any PAA-AOP. As with any oxidant, higher initial concentrations of PAA (and coexistent H_2_O_2_) would accelerate the degradation performance, but an excess might scavenge the generated radicals (and decrease the absorbed UV-photon flux from the active species in irradiated systems), thereby inhibiting CEC degradation [28]. Furthermore, generated or already present acetic acid and acetate might scavenge HO^•^ (k = 1.6 × 10^7^ M^−1^ s^−1^ and 8.5 × 10^7^ M^−1^ s^−1^, respectively [53]) and the generated organic radicals (^•^CH_2_C(O)O^−^ and ^•^OOCH_2_C(O)O^−^) are probably poorly reactive against CECs [54]. ROS scavenging also occurs with catalyst excess, apart from representing unnecessary additional costs. Finally, pH might affect differently the process: alkaline solutions are usually more favourable towards CEC abatement by UV/PAA, since peracetate has higher photolysis quantum yields than the peracetic acid [55,56], but iron could precipitate in Fe/PAA systems or alter the surface charge/potential of heterogeneous catalysts, which could reduce interaction with PAA [23].

### 2.4 Peracetic acid activation by transition metals

In the past few years, activation of PAA with transition metals was assumed to behave in a similar way as with Co(II), the former firstly reported by Bawn & Williamson (1951): initial activation by transition metal, generic M^n+^, firstly produces homolytic O–O scission (R17) and the catalytic cycle closes with the reaction of oxidised M^(n+1)+^ with another molecule of PAA, producing CH_3_C(O)OO^•^ and the reduced metal species (R18) [57].

R17 M^n+^ + CH_3_C(O)OOH → M^(n+1)+^ + CH_3_C(O)O^•^ + OH^−^

R18 M^(n+1)+^ + CH_3_C(O)OOH → M^n+^ + CH_3_C(O)OO^•^ + H^+^

**Fig. 1:**
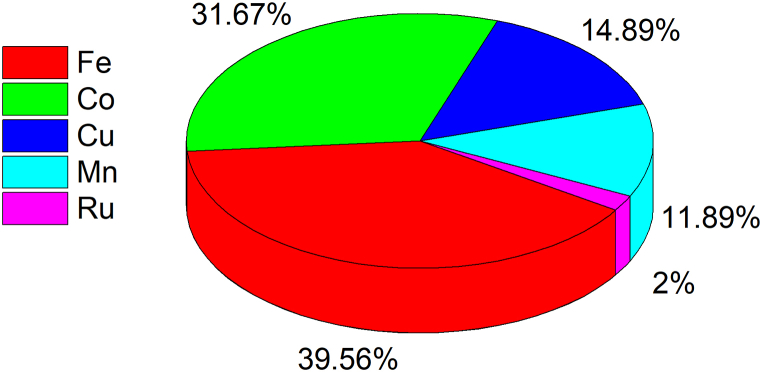
Corresponding percentage of each metal for advanced oxidation processes based on peracetic acid activation exclusively by transition metals. Searches terms: “peracetic acid” AND “activation” AND “metal (e.g., Co, Fe, Mn, *etc.*)” (source: Scopus, last access 5^th^ September 2023).

According to the available literature, the most common metals employed for PAA activation are Fe (40% of total publications), Co (32%), Cu (15%), Mn (12%) and, recently, Ru (2%) (Fig. 1). PAA activation by transition metals has been thoroughly studied in the past for the synthesis of specific compounds [58] or the delignification of pulp [59]. Similarly to PMS or PDS, the highest catalytic performance for PAA activation is obtained with Co-based catalysts rather than with any other transition metal [23,60], which explains the high number of studies applying Co/PAA.

Contrarily to iron, it is usually reported that Co/PAA (or Co/PDS or PMS) is not highly pH-dependent and shows comparable CEC removal rates at pH 5 or 9 [[61], [62], [63]]. This might be explained by three facts: *(i)* E°(Co(III)/Co(II)) = 1.81 V vs. NHE, thus Co(II) (highly soluble) oxidation by O_2_ is thermodynamically less favoured than Fe(II) (E°(Fe(III)/Fe(II)) = 0.77 V vs. NHE); *(ii)* although Co(III) solubility in water is extremely low (K_sp_ ≈ 1 × 10^−50^ for Co(III) (oxy)hydroxides [64]), Co(III)-oxides exhibit higher catalytic activity against PAA decomposition than Fe(III)-oxides [65,66], and *(iii)* the Fenton-like reaction between Co(III) and PAA is considerably faster than that of Fe(III) [23]. Besides higher catalytic activity, there is negligible HO^•^ formation within Co/PAA, which is thus very useful to assess the reactivity of RO^•^ alone [67]. Nevertheless, opposed to Fe, Co is highly toxic and it is a critical element with few manufacturers (the main producer is the Democratic Republic of Congo, with 63% of the world's production), not to mention the complex and dramatic issues related to its extraction [68]. Therefore, the use of Co compounds in environmental remediation is discouraged.

## 3 Peracetic acid activation by iron-based processes

**Fig. 2:**
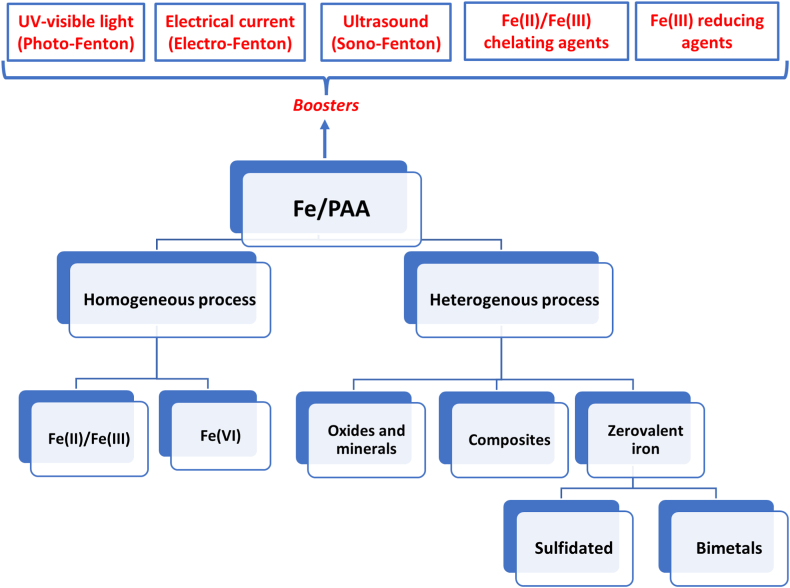
Summary of peracetic acid activation processes based on iron compounds.

PAA activation by iron can occur in homogeneous and heterogeneous systems, and it is enhanced in presence of iron chelating agents, light, electrical current, or ultrasound. Fig. 2 shows a summary of the strategies proposed up to date in Fe/PAA, which will be described in this section.

### 3.1 Iron aquatic speciation

Although iron is the fourth most abundant element in the Earth's crust, it can be detected only at negligible concentrations in natural waters due to the fast oxidation of Fe(II) at neutral pH (k(Fe(II)/O_2_) ≈ 20 M^−1^ s^−1^ [69], R19) and the subsequent formation of insoluble iron (oxy)hydroxides (i.e., Fe_2_O_3_, FeO(OH), and Fe(OH)_3_, logK_s__p_ ≈ −40 [70]) due to Fe(III) fast hydrolysis (R20). Noteworthy, charge neutralisation of colloids by formed iron-species and incorporation of impurities in respective amorphous (oxy)hydroxides is, at the same time, the basis of the use of iron salts as flocculant/coagulant agents in DWTPs and UWTPs [71]. Table 2 summarises the respective equilibrium constants of Fe(II) and Fe(III).

**Table 2:** Reported stability constants for ferric aquo-complexes. Reported values are at T = 25 °C and ionic strength 0.1 mol kg^−1^.

Reaction	logβ	References
Fe^2+^ + H_2_O → Fe(OH)^+^ + H^+^	−9.7	[149,168]
Fe^3+^ + H_2_O → Fe(OH)^2+^ + H^+^	−2.6	
Fe^3+^ + 2 H_2_O → Fe(OH)_2_^+^ + 2 H^+^	−6	
Fe^3+^ + 3 H_2_O → Fe(OH)_3(aq)_ + 3 H^+^	−14	
Fe^3+^ + 4 H_2_O → Fe(OH)_4_^-^ + 4 H^+^	−22	
2 Fe^3+^ + 4 H_2_O → Fe_2_(OH)_2_^4+^ + 6 H^+^	−2.9	
Formation of Fe(OH)_3(s)_, Fe_2_O_3(s)_, FeOOH_(s)_	39 (-logKsp)	

The optimal pH conditions for any Fenton-based treatment are achieved at 2.7 < pH < 3.3, which is attributed to three reasons: *(i)* no formation of iron oxides (relevant at pH ≥ 4), *(ii)* Fe(II) occurs significantly as Fe(OH)^+^ and Fe(OH)_2(aq)_ species, which have higher kinetic rate constants with H_2_O_2_ than Fe^2+^ [12] (a fact not explored yet for PAA), and *(iii)* greater abundance of photoactive species, such as Fe(OH)^2+^ (R20) and Fe(HO_2_)^2+^ (R21, intermediate from R2 [72]). By extension, it can be expected the formation of Fe(CH_3_C(O)OO)^2+^ as plausible metastable complex between Fe(III) and PAA (R22). Ferric species, including Fe(OH)^2+^, Fe(HO_2_)^+^, and Fe(CH_3_C(O)OO)^2+^ are discussed in section 3.2.4.

R19 4 Fe^2+^ + 4 H^+^ + O_2_ → 4 Fe^3+^ + 2 H_2_O

R20 [Fe(H_2_O)_6_]^3+^ ⇆ H^+^ + [Fe(H_2_O)_5_(OH)]^2+^ ⇆ H^+^ + [Fe(H_2_O)_4_(OH)_2_]^+^ ⇆ … ⇆ Fe_2_O_3(s)_ + FeO(OH)_(s)_ + Fe(OH)_3(s)_ + others

R21 Fe^3+^ + H_2_O_2_ ⇆ Fe(HO_2_)^2+^ + H^+^

R22 Fe^3+^ + CH_3_C(O)OOH ⇆ Fe(CH_3_C(O)OO)^2+^ + H^+^

### 3.2 Homogeneous activation by iron

#### 3.2.1 Dark Fenton process: ferrous and ferric ions

A revised mechanism of PAA with Fe(II) has been proposed recently (reactions R23-R27 [24]). It was observed that besides generating CH_3_C(O)O^•^ (R23), Fe(II) could lead to a classic Fenton reaction with PAA (R24), generating HO^•^ as well as FeO^2+^ upon reduction of PAA to HAc (R25); HO^•^ and FeO^2+^ are also formed by coexistent H_2_O_2_ by reactions R1 and R9, respectively. The catalytic loop is closed by reaction of Fe(III) with another molecule of PAA, generating Fe(II) and CH_3_C(O)OO^•^ (R26). The subsequent chain reaction by the formed HO^•^ and RO^•^ is later described in section 3.2.4, R29-R36.

Reactions R23-R25 and R26 (the new Fenton and Fenton-like reactions, respectively) are 2–3 orders of magnitude faster than those with H_2_O_2_ (R1, R2), with k ≈ (0.1–1) × 10^5^ M^−1^ s^−1^ for Fe(II)/PAA and k ≈ 3 M^−1^ s^−1^ for Fe(III)/PAA. One of the reason of these differences could be the aforementioned lower O–O energy of PAA compared to H_2_O_2_.

R23 Fe^2+^ + CH_3_C(O)OOH → Fe^3+^ + CH_3_C(O)O^•^ + OH^−^ ; k = (0.16–1.10) × 10^5^ M^−1^ s^−1^

R24 Fe^2+^ + CH_3_C(O)OOH → Fe^3+^ + CH_3_C(O)O^−^ + HO^•^ ; k as R23

R25 Fe^2+^ + CH_3_C(O)OOH → FeO^2+^ + CH_3_C(O)OH ; k as R23

R26 Fe^3+^ + CH_3_C(O)OOH → Fe^2+^ + CH_3_C(O)OO^•^ + H^+^ ; k = 2.72 M^−1^ s^−1^

R27 2 FeO^2+^ + CH_3_C(O)OOH → 2 Fe^3+^ + CH_3_C(O)O^−^ + OH^−^ + O_2_

A very interesting experimental evidence is the fast degradation of organic molecules (methylene blue, naproxen, and bisphenol-A, 15 μM each) with Fe(II)/PAA at pH 7.0, producing 50–70% pollutant removal in 2 h [24]. This is not possible in classic Fe(II)/H_2_O_2_ without iron chelating agents. CEC degradation by Fe(II)/PAA follows two kinetic stages: fast degradation in the first minutes (e.g., 80 and 40% removal of naproxen in 1 min at pH 3.0 and 7.0, respectively) involving the ROS generated by fast reactions with Fe(II) (R23-R25), followed by a much slower process involving PAA and Fe(III), R26 (in 30 min, >95% removal of naproxen at pH 3.0, 65% at pH 7.0). Moreover, comparable PAA decomposition rates were reported in the 3.0–6.0 pH range, obtaining k(Fe(II)/PAA) = (1.10 ± 0.02) × 10^5^ M^−1^ s^−1^ at pH 3.0, and (6.69 ± 0.01) × 10^4^ M^−1^ s^−1^ at pH 6.0. PAA decomposition was slightly lower at pH 7.0 ((5.01 ± 0.01) × 10^4^ M^−1^ s^−1^), and decreased further at pH 8.0 (k(Fe(II)/PAA) = (1.56 ± 0.01) × 10^4^ M^−1^ s^−1^).

The above comments suggest that even in slightly alkaline conditions (pH 8), Fe(II)/PAA can still oxidize pollutants effectively in the absence of iron chelating agents, differently from Fe(II)/H_2_O_2_ where the efficiency drastically falls [12]. On the one hand, at pH 8.0, the reaction between Fe^2+^ and H_2_O_2_ (k(Fe(II)/H_2_O_2_) ≈ 60 M^−1^ s^−1^) is comparable to that of Fe^2+^ with O_2_ (k(Fe(II)/O_2_) ≈ 20 M^−1^ s^−1^, see section 3.1), the latter still several orders of magnitude slower than that of Fe(II) with PAA (k(Fe(II)/PAA) ≈ 5 × 10^4^ M^−1^ s^−1^). On the other hand, the Fenton-like step is approximately 300–3000 times faster by Fe(III)/PAA than by Fe(III)/H_2_O_2_. Therefore, Fe^2+^ regeneration is also more advantageous when employing Fenton processes based on PAA compared to classic H_2_O_2_.

Noteworthy, the aforementioned work did not mention the plausible formation of Fe-PAA complexes within the proposed mechanism, although several studies have reported the formation of metastable metal-peroxy intermediates (e.g., Fe(HO_2_)^2+^, R21) [12,73,74]. In this sense, the formation of these kinds of Fenton-like intermediates was reported during the Co(II)/PAA process, estimating that Co(CH_3_C(O)OO)^+^ is the main reactive specie [75]. Therefore, it is highly probable that a similar way of action occurs in the Fe(II)/PAA process. The formation of Fe(CH_3_C(O)OO)^2+^ was speculated in section 3.1, R22, whose equilibrium constant should be higher than that with H_2_O_2_ (logK(Fe(HO_2_)^2+^) ≈ 9 [76]) due to the possibility of formation of a five-centre complex by CH_3_C(O)OO^−^ vs. single coordination mode of HO_2_^−^. Furthermore, because of the unique structure and composition of PAA, the chelation impact of the either generated or already present Ac^−^ (logK(Fe(CH_3_C(O)O)^2+^) = 3.5 [77]) or other intermediates was neither discussed, nor considered in the mechanism proposed by Kim and co-workers. In fact, apart from the former work, there are no other studies analysing the mechanism of PAA activation by iron. Further studies analysing kinetic rate constants and reactions involved are thus needed, not only to assess the reproducibility of the data reported in the thorough work of Kim et al., but also to calculate the stability constant of the Fe(CH_3_C(O)OO)^2+^ complex, and analyse the plausible influence of iron chelation by the other generated organic by-products (e.g., acetate).

The effect of the starting speciation of iron (i.e., Fe(II) or Fe(III)) with H_2_O_2_ and PAA was evaluated towards the decolorization of methylene blue (MB). In optimal Fenton conditions (pH 3.0 in demineralised water), MB degradations with Fe(II)/H_2_O_2_ or Fe(II)/PAA did not exhibit significant differences, but when starting from Fe(III), the dye absorbance decay was 3 times faster with PAA than with H_2_O_2_ [78]. These results are in agreement with the kinetic rate constants of R26.

Besides pharmaceuticals, as uncommon CEC target, p-arsanilic acid (an organoarsenic compound) was degraded by PAA-Fenton process in different conditions [79]. Regarding pH, analogous observations as for previous works were reported: degradation was evidently fast in acidic conditions, but also observed at neutral-alkaline ones (e.g., p-arsanilic acid removal by 50% in 5 min at pH 7). Among the generated by-products, As^V^O_4_^3−^ (less toxic and mobile than As^III^O_3_^3−^) and nitrasone ((p-nitrophenyl)arsonic acid) were detected. The inorganic pollutant was eliminated from water by co-precipitation on generated solid ferric (oxy)hydroxides, which are well known to have high affinity for arsenic adsorption [80]. Authors also reported that PAA alone was able to oxidize As(III) into As(V), which was not possible by H_2_O_2_ alone. This might be beneficial when iron is already precipitated (therefore, with scarce activation of PAA or H_2_O_2_) but when residual PAA still occurs at the same time, which is not possible with H_2_O_2_.

Wastewater sludge treatment by Fe-PAA processes was also investigated, and removal of water from treated sludge exhibited better performance when using Fe(II)/PAA at neutral pH compared to Fe(II)/H_2_O_2_; Fe(II)/PAA was also less affected by pH variations [81]. However, Fe(II)/PAA caused higher organic matter degradation and the generated Fe(III) played a major role in the re-flocculation process; therefore, stronger aggregation was observed in the sludge treated with Fe(II)/H_2_O_2_.

Based on the above-mentioned statements, Fe(II,III)/PAA generally exhibits better performances toward CEC degradation at every tested pH value compared to Fe(II,III)/H_2_O_2_ (or equal at pH 3). However, none of the cited works monitored the TOC concentration along the process, a parameter for which classical Fenton probably shows better results. Furthermore, results are mostly focused on the first seconds of the reaction, and the kinetics of the resulting by-products and TOC evolution at the hour-scale is scarce. Studies analysing CECs and their degradation intermediates at neutral pH at longer times, as well as TOC kinetics, should be mandatory for any study employing Fe/PAA to obtain further details into the overall treatment performance.

#### 3.2.2 Enhancing Fenton process at neutral pH: use of chelating and reducing agents

The main drawback of Fenton-based processes is the fast hydrolysis of Fe(III) to insoluble iron oxides at circumneutral pH, which hinders the reaction and generates iron sludge. Acidification pre-treatment is simple and economic when dealing with ultra-pure water and/or demineralised water; however, the amount of required acid drastically increases in real water due to its alkalinity (i.e., the buffer capacity of the CO_2(aq)_/HCO_3_^−^ couple). Besides, the final effluent must be re-neutralised with alkali dosages before final discharge, to comply with water quality standards. Nevertheless, commonly used acids such as H_2_SO_4_ are not expensive and acidification eliminates HCO_3_^−^ as CO_2(g)_, hence getting rid of the main inorganic HO^•^ scavenger. Besides acidification, consecutive iron dosages are reported to be more efficient than adding the same quantity at once at mild pH conditions [82]. The most efficient method to drive Fenton processes at neutral pH is the use of iron chelating (e.g., EDDS or NTA) and/or reducing (e.g., hydroxylamine or boron) agents [83]. However, the addition of such reagents implies an overall increase in treatment costs and plausible ROS scavenging by the added substances, which could also be toxic, non-biodegradable, or generate hazardous by-products.

##### 3.2.2.1 Iron chelating agents

**Fig. 3:**
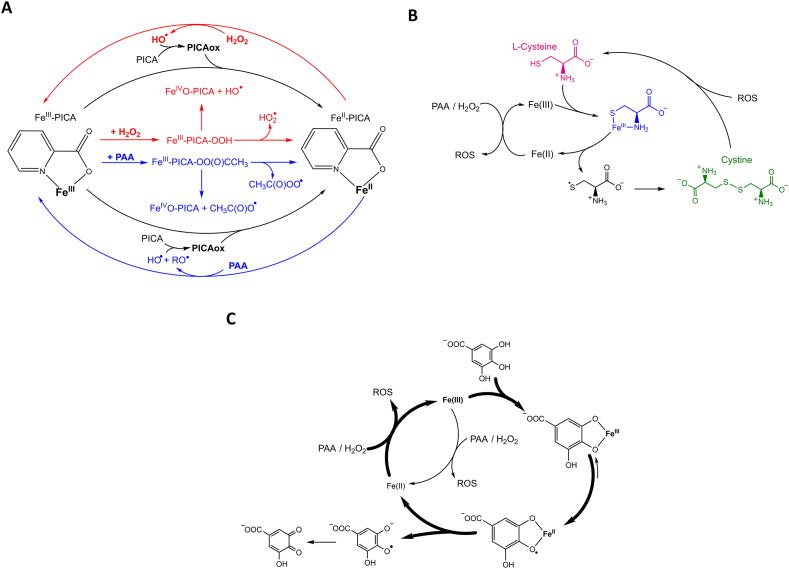
Scheme of different iron chelating agents reported with Fe/PAA and their mechanism of iron cycle enhancement: A) PICA [73,84], B) l-cysteine [86,87], and C) gallic acid [156].

Three iron chelating agents have been studied to improve PAA-Fenton processes: picolinic acid, l-cysteine, and gallic acid. A scheme with their respective operative mechanisms is reported in Fig. 3.

Kim et al. (2022) compared the oxidation of MB by Fe(III)/PAA in presence of different chelating agents: EDTA (ethylenediaminetetraacetic acid), NTA (nitrilotriacetic acid), citric acid, and PICA (picolinic acid), obtaining outstanding dye decolorization rates only with the latter. At the following initial conditions: pH 5.0, [MB] = 15 μM, [Fe(III)] = 50 μM, [PAA] = 500 μM, and 125 μM of chelating agent, the model contaminant was degraded by approximately 90% in 4 min when employing PICA, while degradation with the other ligands was negligible in the same time scale. PICA efficiency was also compared with structurally similar compounds: nicotinic acid, proline, and dipicolinic acid. Nicotinic acid also has a pyridine ring but with the carboxyl substituent in position 3 instead of 2, thereby acting as a monodentate ligand; it exhibited scarce dye oxidation in the first 4 min. In the case of proline, although the distance between the carboxylic and nitrogen moieties allows formation of a five-centred chelate ring with Fe(III), dye removal was considerably slower. Finally, with dipicolinic acid (with an additional carboxylic moiety in position 2 of the pyridine ring) 15% degradation in 4 min was obtained, thus being the second most efficient tested chelating agent. Still, dipicolinic acid carried out the Fenton-PAA process 6 times more slowly than picolinic acid. Fe(III)/PICA/PAA was also employed to degrade other CECs (such as naproxen, carbamazepine, and diclofenac), each at 15 μM concentration at pH 7.0, obtaining >50% degradation in 10 min for all of them [84].

The performance of PICA might be related to both iron-chelating capacity and Fe(III) reduction, which would accelerate iron cycling (Fig. 3A). In fact, it was previously reported that the use of PICA in Fe(III)/H_2_O_2_ at pH 5.0 generated 7–12 times more HO^•^ than the system without ligand at pH 3.0 [73]. In addition, it was also suggested that the main reactive species contributing to CEC degradation by Fe(III)/PICA/PAA was high-valent iron rather than HO^•^. This is in agreement with Farinelli et al. (2020), who reported that iron chelation enhances CECs degradation by FeO^2+^ instead of HO^•^, the latter being the dominant oxidative species without chelating agents [85].

Similarly to PICA, l-cysteine is an interesting Fe(III) chelating-reducing agent and it is reported to act as co-catalyst (Fig. 3B). The S–S bond of cystine (formed after Fe(III) reduction) is in fact cleaved by HO^•^, regenerating the original cysteine [86]. Fe(III)/L-cysteine/PAA was applied to degrade the sulfonamide antibiotic, sulfamethoxazole (SMX) [87]. At pH 5.5, addition of 0.25 mM l-cysteine accelerated degradation of 10 μM SMX and achieved 85% removal in only 5 min, compared to 20% removal in 1 h without the chelating agent ([Fe(III)] = [PAA] = 0.5 mM in both cases). Although the employed concentration of iron in the mentioned study was relatively high, the most remarkable aspect was the high degradation efficiency in alkaline conditions, which was extended up to pH 11.

Alongside synthetic chelating agents, ubiquitous DOM fractions were also reported to enhance Fenton processes at neutral pH due to polyphenol moieties [15]. Fe(III)/PAA/gallic acid was used to degrade 25 μM BPA at pH 3–9, employing concentrations of gallic acid, Fe(III), and PAA of 25, 50, and 100 μM, respectively. Optimal pH conditions were obtained at pH 4–5 (with pseudo-first order BPA degradation rate constant of 0.16 min^−1^), followed by pH 7.0 (k_BPA_ = 0.14 min^−1^), pH 3.0 ≈ pH 8.0 (k_BPA_ = 0.09 min^−1^), and pH 9.0 (k_BPA_ = 0.075 min^−1^) [88]. Outside the 4–7 pH range, iron was not present as soluble complexes (at pH < 4, protonated polyphenols are unable to bind iron) or precipitated as oxides (pH > 8), in agreement with other works [89]. The positive role of phenolic moieties acting as iron chelating/reducing agents (Fig. 3C) was invoked to explain the faster degradation, by Fe(III)/PAA, of BPA compared to other pollutants: the fast homolytic scission of BPA into two p-ethylphenol molecules generated polyphenolic by-products with chelating properties [88].

##### 3.2.2.2 Fe(III) reducing agents

Up to now, only ABTS (2,2′-azino-bis(3-ethylbenzothiazoline-6-sulfonate)) and hydroxylamine have been used as reducing agents in Fe/PAA treatments. As heterogeneous co-catalysts, boron and MoS_2_ are reported.

ABTS is usually employed as indicator for the spectrophotometric determinations of oxidants [90] and was proposed as a novel electron shuttle to enhance the PAA-Fenton reaction. At pH 3.0, [PAA] = 100 μM and 1 μM Fe(II), the addition of ABTS 25 μM enhanced the degradation of diclofenac and achieved >90% degradation in 30 min, compared to ca. 10% without ABTS. The same conditions were applied at different pH values, observing negligible removals at 30 min at pH 5–9. In this system, ABTS is likely oxidized to ABTS^•+^ by Fe(III) and RO^•^, until the formation of toxic by-products (sulfoxides and sulfones, produced by an undesired parallel reaction). However, it was also observed that instead of HO^•^, RO^•^, or high-valent iron, ABTS^•+^ was the main specie responsible for diclofenac oxidation, regenerating ABTS [91]. Compared to ABTS, NH_2_OH seems to be more efficient and produced 6 times faster diclofenac degradation, with considerable pollutant removal until pH 6.0. However, differently from ABTS, NH_2_OH is consumed and mostly decomposed into N_2_ (but also N_2_O, NO_2_^−^, and NO_3_^−^), making the enhancement difficult to be sustained without constant NH_2_OH addition. Actually, NH_2_OH is a reagent rather than a co-catalyst or an electron shuttle [92,93].

The use of solid reducing agents, such as boron or MoS_2_, has recently attracted interest in the enhancement of Fenton reactions (with PAA, H_2_O_2_, or PMS) as a promising solid co-catalyst that, as such, would have separation and reuse potential and would avoid an additional route of TOC increase, ROS scavenging, or release of oxidation by-products. Amorphous and crystalline boron were compared in a Fe(III)/PAA system for BPA degradation, and only the amorphous form was able to accelerate the iron cycle. A 145-fold kinetic increase at pH 6 was obtained with amorphous B compared to the systems without B, or with crystalline B. BPA degradation after 20 min was negligible with Fe(III)/B_crystalline_/PAA, whereas it amounted to >90% with Fe(III)/B_amorphous_/PAA. The higher electron-donor capacity of amorphous boron was assigned to its markedly lower particle size (higher contact surface) and greater asymmetry of B_12_ icosahedron structure, which favoured the B–B bond cleavage. The continuous formation of interfacial suboxide boron intermediates boosted the reaction, by continuously donating electrons to Fe(III) [94]. Sulfamethazine (SMT) was also degraded by an analogous system with boron powder at pH 3, with analogous enhancement and allowing for at least 4 consecutive co-catalyst reuse cycles with addition of PAA only [95]. Besides boron, MoS_2_ is also known to enhance the PAA-Fenton-like process. MoS_2_ reduces Fe(III) to Fe(II) with parallel oxidation of Mo(IV) to Mo(VI), and it can also activate PAA (or other oxidants) through the same mechanism previously described for transition metals (see R17 and R18). CEC degradation could thus be achieved in 10 min, while scarce degradation was obtained without MoS_2_ [96].

#### 3.2.3 Ferrate(VI)

Up to now, only two articles reported the use of ferrate(VI) and PAA [97,98]. FeO_4_^2−^ (pK_a_ = 7.2) is a strong oxidant, with E° = 2.20 V vs. NHE for the H_3_FeO_4_^+^/Fe^3+^ redox couple and E° = 0.70 V vs. NHE for the FeO_4_^2−^/Fe(OH)_3_ one [99]. The self-decay of FeO_4_^2−^ into highly reactive Fe(IV) and Fe(V) species (such as HFeO_4_^3−^ and HFeO_4_^2−^, respectively) can be promoted by several compounds, such as SO_3_^2−^, NH_2_OH, or carbonaceous materials (e.g., graphene oxide) [100]. Fe(VI)/PAA achieved almost instantaneous degradation of 10 μM carbamazepine (>99% in less than 1 min at pH 9) with [FeO_4_^2−^]/[PAA] ratios between 2 and 4 ([PAA] = 100 μM). With complex water matrices (tertiary WWTP effluent, with pH 7 and 6 mg_C_ L^−1^ total organic carbon, containing a mixture of three CECs at 10 μM each), the degradation percentages reached 87–100% in only 5 min [97]. Surprisingly, PAA does not significantly activate FeO_4_^2−^ (reported bimolecular kinetic rate constant k(Fe(VI)/PAA) = 1.4 ± 0.1 M^−1^ s^−1^), and activation by coexistent H_2_O_2_ is more important (k(Fe(VI)/H_2_O_2_) = 20 ± 1 M^−1^ s^−1^). This was evidenced by the degradation of carbamazepine with Fe(VI)/PAA at pH 7.3 (thus, pKa_Fe(VI)_ ≤ pH < pKa_PAA_), which did not exhibit significant differences with Fe(VI)/H_2_O_2_ (H_2_O_2_ concentration was 38 μM, which is the same as that occurring in the employed 100 μM PAA solutions). Therefore, H_2_O_2_ (not PAA) is mainly responsible for Fe(VI) activation at neutral pH. The enhancing effect of PAA was only noticeable at pH > 8, indicating that the deprotonated PAA form was the specie involved in carbamazepine removal. Authors proposed that Fe(IV)/Fe(V)-OO(O)CCH_3_ complexes could be formed and react with CECs, avoiding the self-decay of Fe(IV)/Fe(V)–OH complexes into Fe(II) and Fe(III). Fe(IV)/Fe(V)-OO(O)CCH_3_ complexes should, in fact, be more reactive than the common Fe(IV)/Fe(V)–OH complexes [98].

Due to the promising results, further studies should be performed to better understand the mechanism behind Fe(VI)/PAA at neutral-alkaline conditions. It could be interesting to explore the combination of ferrate(VI) with an activator (e.g., graphene oxide), to enhance the formation of Fe(IV)/Fe(V) species in the presence of PAA.

#### 3.2.4 Photo-Fenton

Photons in the 200–300 nm range cleave the O–O bond of PAA and can be produced with traditional UVC-UVB lamps, novel light emission diodes (LEDs), or sunlight [26,27,101]. Similarly to H_2_O_2_, deprotonated PAA (PAA^−^, pH > 8.2) is more photolabile than the protonated species. At 254 nm, the molar absorption coefficient of PAA^−^ is 58.9 M^−1^ cm^−1^, compared to 10.0 M^−1^ cm^−1^ for PAA, and the respective photolysis quantum yields are Φ_PAA_- = 2.09 mol E^−1^ and Φ_PAA_ = 1.20 mol E^−1^ [55]. Homolytic O–O cleavage generates HO^•^ and CH_3_C(O)O^•^ from PAA (R28), which react both with PAA (R29-R31) and between themselves (R32-R34) to produce several ROS (HO_2_^•^, O_2_^•−^, and ^3^O) and organic radicals (R^•^, RO^•^). A complete photolytic degradation mechanism of PAA takes into account the role of H_2_O_2_ and HO^•^ scavenging by HAc [54]. After HO^•^, CH_3_C(O)OO^•^ is usually reported as the main reactive species responsible for CEC abatement in UV/PAA systems, followed by CH_3_C(O)O^•^. Actually, CH_3_C(O)O^•^ is rapidly self-decomposed into ^•^CH_3_ (R35) that is a weak oxidant and reacts rapidly with dissolved oxygen to produce CH_3_OO^•^ (R36), eventually decomposed into HO_2_^•^, O_2_, formaldehyde, and methanol [23,54].

R28 CH_3_C(O)OOH + hν → CH_3_C(O)O^•^ + ^•^OH ; Φ_254_ = 0.9–1.20 mol Ein^−1^

R29 CH_3_C(O)OOH + HO^•^ → CH_3_CO^•^ + H_2_O + O_2_ ; k = 1 × 10^9^ M^−1^ s^−1^

R30 CH_3_C(O)OOH + HO^•^ → CH_3_C(O)OO^•^ + H_2_O ; k =1 × 10^9^ M^−1^ s^−1^

R31 CH_3_C(O)OOH + CH_3_C(O)O^•^ → CH_3_C(O)OO^•^ + CH_3_C(O)OH ; k = (0.01–1) × 10^7^ M^−1^ s^−1^

R32 CH_3_C(O)O^•^ + HO^•^ → CH_3_C(O)OOH ; k = 1 × 10^9^ M^−1^ s^−1^

R33 2 CH_3_C(O)O^•^ → 2 (CH_3_C(O)O)_2_ ; k = 1 × 10^9^ M^−1^ s^−1^

R34 2 HO^•^ → H_2_O_2_ ; k = 5.5 × 10^9^ M^−1^ s^−1^

R35 CH_3_C(O)O^•^ → ^•^CH_3_ + CO_2_ ; k = 2.3 × 10^5^ s^−1^

R36 ^•^CH_3_ + O_2_ → CH_3_OO^•^ ; k = 4.7 × 10^9^ M^−1^ s^−1^

**Fig. 4:**
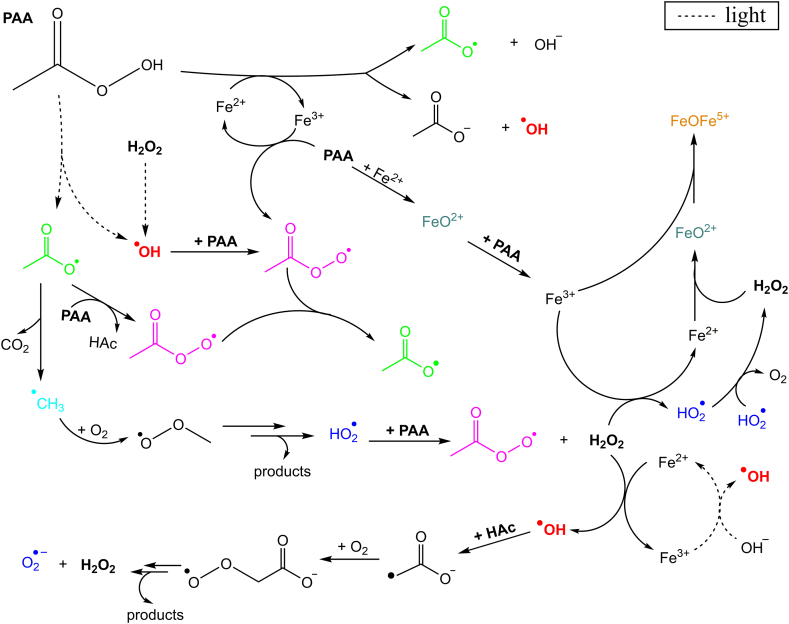
Scheme of PAA-photo-Fenton mechanism based on the results published by Zhang et al. (2020) and Kim et al. (2019) [24,54].

Pollutant abatement with photo-Fenton processes is faster than in analogous dark conditions, also in the presence of PAA, related to the following parallel reactions: *(i)* photogeneration of Fe^2+^ and HO^•^ through Fe(OH)^2+^ photolysis (R37) or of L^•^ instead of HO^•^, when L is a ligand different from OH^−^ (R38), *(ii)* activation of H_2_O_2_ and PAA by direct O–O photolytic scission (R39 and R28, respectively), and *(iii)* (in)direct photodegradation of CECs [56]. A scheme of PAA-photo-Fenton reaction is shown in Fig. 4. Decomposition of Fe(HO_2_)^2+^ by light is sometimes proposed in photo-Fenton mechanism (R40) [12], therefore, by extension, it is plausible that Fe(CH_3_C(O)OO)^2+^ undergoes photolysis too (R41).

R37 Fe(OH)^2+^ + hν → Fe^2+^ + HO^•^

R38 Fe^3+^-L_n_ + hν → Fe^2+^-L_n-1_ + L^•^

R39 H_2_O_2_ + hν → 2 HO^•^

R40 Fe(HO_2_)^2+^ + hν → Fe^2+^ + HO_2_^•^

R41 Fe(CH_3_C(O)OO)^2+^ + hν → Fe^2+^ + CH_3_C(O)OO^•^

Several recent studies have reported the use of UV light to enhance Fe/PAA treatments. Wang et al. (2021) studied the degradation of 1 μM triclosan at initial pH 3.5 by UV/PAA, Fe(II)/PAA, and UV/Fe(II)/PAA, reporting removal rate constants of 0.10, 0.46, and 0.59 min^−1^, respectively. PAA-photo-Fenton did not show high pH-dependence, with only slightly slower removal rates in neutral conditions than in acidic ones (at 5 min, approximately 85% removal at pH 7.0 vs. 95% in the same time frame at pH 3.5). Moreover, negligible effect of Cl^−^ was observed up to [Cl^−^] = 20 mM. For concentrations of HCO_3_^−^ ≤ 10 mM and NOM ≤5 mg L^−1^, the pollutant removal rates marginally decreased; in contrast, with 10 mM NO_3_^−^ the removal was slightly faster [102]. Similar studies, analysing operational parameters and the effect of water constituents, were recently reported employing PAA-photo-Fenton with ferric salts [103] and sunlight for irradiation instead of UV lamps [78], suggesting that this process has great potential.

Ghanbari et al. (2021) studied the efficiency of different transition metals to activate PAA under LED UVC. The reported acetaminophen (20 mg L^−1^ = 132 μM) removal rates followed the order: Fe(II) > Cu(II) ≥ Co(II) > Ag(I) ≥ Mn(II) > Fe(III), under conditions of pH 3.0, [PAA] = 3 mM and 0.5 mM of the tested metal catalyst [26]. In addition, use of LED instead of conventional UVC lamps brings several advantages: lower energy consumption, no need of pre-heating, and longer lamp lifetimes. Furthermore, the mentioned work is one of the very few that monitored TOC along the whole experiment, evidencing that this parameter is affected by PAA.

An additional energy-saving frontier alternative is represented by vacuum ultraviolet systems (VUV), which have been applied to the abatement of 10 μM carbamazepine at pH 3–9 by the PAA-photo-Fenton process. In all cases, degradations ≥80% were obtained in only 5 min, employing low concentrations of PAA (50 μM) and Fe(II) (10 μM) [104]. This outstanding performance is also due to the well-known fact that 185 nm VUV light can photolyse pollutants, as well as H_2_O into HO^•^ and H^•^ (or, with a lower quantum yield, produce H^+^ and solvated electrons), and O_2_ into O [105].

Apart from CEC degradation, PAA-photo-Fenton has been proposed as a peroxidation process prior to ultrafiltration to mitigate the membrane fouling caused by NOM, thereby improving the membrane flux by a factor of ca. 2 and decreasing both reversible and irreversible fouling [106].

#### 3.2.5 Electro- and sono-Fenton

In addition to light, electrical current (EC) and ultrasound (US) are the other two possibilities for extending the Fenton process efficiency without iron-chelating or ferric-reducing agents. Both EC [107] and US [108] are able to rapidly decompose PAA, generating HO^•^ and RO^•^.

When applying EC, anodic oxidations include the plausible degradation of the CEC itself, generation of CH_3_C(O)OO^•^ (R42), and oxidation of water into HO^•^ (R43), whereas the cathodic reactions consist in the *in situ* electrogeneration of H_2_O_2_ (R44), the reduction of Fe(III) (R45), and the generation of HO^•^ and CH_3_C(O)O^•^ from PAA (R46 and R47, respectively.) A Fenton reaction without external H_2_O_2_ addition is thus carried out [109].

Yuan et al. (2022) employed electro-Fenton-PAA (EC/Fe(II)/PAA) to degrade MB with a current density of 15 mA cm^−2^. As usually observed with Fenton processes, MB decolorization was faster at pH 3 (>99% in 1 h) than at pH 7 (50% in 1 h). When comparing different processes, at pH 3 dye decolorization rate followed the order: EC/Fe(II)/PAA ≫ EC/PAA > EC = EC/Fe(II) > Fe(II)/PAA, demonstrating the synergistic effect towards PAA activation, and also indicating the strong effect of electro-decomposition of PAA alone [110].

R42 CH_3_C(O)OOH → CH_3_C(O)OO^•^ + e^−^

R43 H_2_O → HO^•^ + e^−^ + H^+^

R44 O_2_ + 2 H^+^ + 2 e^−^ → H_2_O_2_

R45 Fe^3+^ + e^−^ → Fe^2+^

R46 CH_3_C(O)OOH + e^−^ → CH_3_C(O)O^•^ + OH^−^

R47 CH_3_C(O)OOH + e^−^ → CH_3_C(O)O^−^ + HO^•^

As far as PAA-sono-Fenton is concerned, US (20–2000 kHz waves) alone is able to decompose water (sonolysis) into HO^•^ and H^•^ (R48), and dissolved oxygen into O^•^ (R49), the last two leading to further formation of HO^•^ (R50). Therefore, similarly to VUV (previously mentioned), US alone generates an oxidative environment able to abate many CECs. When adding PAA to the system, US easily induces cleavage of O–H, O–O, and C–C bonds, generating HO^•^ (R51), CH_3_C(O)O^•^ (R52), and CH_3_^•^ (R53), respectively [108]. Such excess of HO^•^ leads to H_2_O_2_ (R34), which later on gets activated by iron ions when carrying the sono-Fenton process (R1 and R2), as well as an accelerated Fenton-like reaction due to the Fe(HO_2_)^2+^ complex decomposition by US (R54), analogous to that happening with light (R40) [111,112]. Bhasarkar et al. (2013) investigated the degradation of dibenzothiophene and toluene (as model sulfur and gasoline hydrocarbon emission gases produced in vehicles combustion) by wet flue gas desulfurization systems (reaction R55 was not proposed in the work, but it is likely to happen based on existing literature) [113]. The same experimental approach but without US (i.e., Fe(II)/PAA) was proposed for the removal NO and SO_2_ gases by other authors [114,115]. No works on US/Fe(II)/PAA for water treatment have been published yet.

R48 H_2_O + US → HO^•^ + H^•^

R49 O_2_ + US → 2 O^•^

R50 H_2_O + O^•^ → 2 HO^•^

R51 O_2_ + H^•^ → O^•^ + HO^•^

R52 CH_3_C(O)OOH + US → CH_3_C(O)O^•^ + HO^•^

R53 CH_3_C(O)O^•^ + US → CH_3_^•^ + CO_2_

R54 Fe(HO_2_)^2+^ + US → Fe^2+^ + HO_2_^•^

R55 Fe(CH_3_C(O)OO)^2+^ + US → Fe^2+^ + CH_3_C(O)OO^•^

### 3.3 Heterogeneous systems

Differently from the homogeneous Fenton reactions, the use of solid iron-containing materials such as iron oxides, iron–composites, and zerovalent iron usually allows for a reduction of the amount of iron sludge in the final effluent, as well as for easy separation of the iron-catalyst through magnetic fields (when applicable) followed by plausible reuse.

#### 3.3.1 Zero-valent iron (ZVI)

ZVI has been thoroughly applied in combination with H_2_O_2_ [116], PMS [117], or PDS [118]. It allows for a controlled and constant release of Fe(II) (R56 and R57), *in-situ* formation of H_2_O_2_ (R58), and easy Fe(III) recycling (R59). As drawbacks, it consumes oxidant (R60 and R61) and also dissolved oxygen, requiring higher oxidant concentrations compared to iron salts and producing anaerobic conditions. This is optimal to have high Fe(II) concentrations even at neutral pH, but a drawback for ROS generation [119]. Moreover, activation pre-treatments (e.g., ultrasound or acid washings) or conditions favouring ZVI corrosion are usually needed to remove the surface-oxide passivation layer, with the consequence that efficient CEC degradation is usually observed under acidic conditions [120,121].

R56 Fe^0^ + 2 H^+^ → Fe^2+^ + H_2_

R57 2 Fe^0^ + O_2_ + 4 H^+^ → 2 Fe^2+^ + 2H_2_O

R58 Fe^0^ + O_2_ + 2 H^+^ → Fe^2+^ + H_2_O_2_

R59 Fe^0^ + 2 Fe^3+^ → 3 Fe^2+^

R60 Fe^0^ + H_2_O_2_ → Fe^2+^ + 2 OH^−^

R61 Fe^0^ + CH_3_C(O)OOH + 2 H^+^ → Fe^2+^ + CH_3_C(O)OH + H_2_O

Compared to H_2_O_2_, the use of ZVI/PAA is reported to be significantly more efficient at neutral pH. A recent work studying the removal of 10 μM tetracycline at pH 6.0 with ZVI nanoparticles showed almost 7 times faster removal with PAA than H_2_O_2_, and CH_3_C(O)OO^•^ was the main radical contributing to pollutant oxidation [122]. Comparable differences were obtained in another study degrading spiramycin at pH 4.0: ca. 30% degradation in 20 min with ZVI/H_2_O_2_, while ZVI/PAA produced 80% degradation [123].

**Fig. 5:**
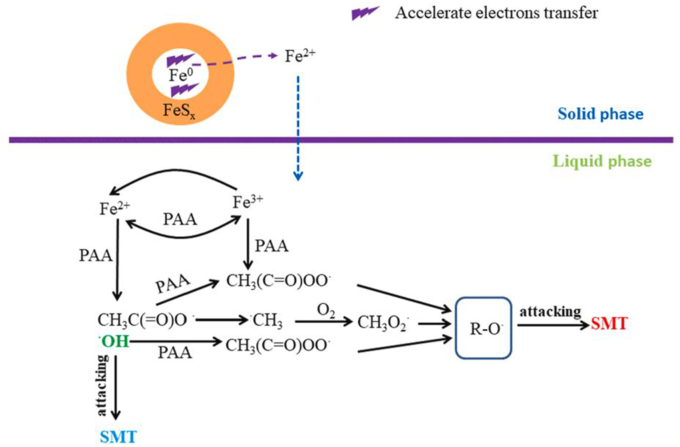
Proposed mechanism of PAA activation by S-ZVI, reprinted with permission from Elsevier, Y. Pan et al. (2021) [126].

Apart from reducing particle size, modification of ZVI by sulfur (sulphidation, S-ZVI) is a simple and inexpensive method to significantly increase ZVI reactivity. Indeed, iron sulphides have higher electron conductivity than iron oxides-hydrates, thereby accelerating Fe(III) reduction and decreasing surface passivation [124] (see Fig. 5). Coherently, Pan et al. (2021) reported that S-ZVI powder allowed for almost complete SMT degradation in 60 min with 100 μM PAA, whereas a *plateau* was obtained after the first 10 min with non-sulfidated ZVI powder. Comparable results were reported when employing S-ZVI microparticles, obtaining complete degradation of a mixture of 6 CECs within 10 min in neutral conditions and maintaining the degradation efficiency for at least 7 cycles [126].

As bimetals containing iron, activation of PAA by Co^0^–Fe^0^ [127] and Cu^0^–Fe^0^ [128,129] have been reported, exhibiting comparable performances. In the first case, Co^0^ could significantly extend the pH tolerance of ZVI and produce fast CEC removal in both acidic and alkaline conditions, due to outstanding PAA decomposition by Co(II)/Co(III). Moreover, Co(III) was reported to enhance ZVI microparticles corrosion, improving PAA activation, while Co^0^ could also reduce Fe(III) (E°(Co(II)/Co(0)) = −0.3 V vs NHE) and close the iron cycle. As a more environmentally friendly alternative, Cu^0^-ZVI triggers PAA decomposition by Cu(I)/Cu(II) cycling, extending the pH range where ZVI is efficient in the Fenton-PAA reaction. Moreover, Cu(I) can reduce Fe(III) (E°(Cu(II)/Cu(I)) = 0.17 V vs NHE), differently from Co(II) (E°(Co(III)/Co(II)) = 1.8 V vs NHE).

#### 3.3.2 Ferric and ferrous materials

##### 3.3.2.1 Sulfur based

In line with the advances obtained with sulphidated-ZVI mentioned above, iron sulphides, FeS and FeS_2_ (pyrite), have been successfully applied to drive heterogeneous Fenton reactions with different oxidants. Yang et al. (2022) compared the degradation efficiency of 10 μM SMX by PDS, PMS, and PAA (100 μM each), activated by FeS (25 mg L^−1^); after 10 min, respective removals of 10, 60, and >99% suggest that PAA/FeS is the most efficient option [130]. The mechanistic study highlighted that the species responsible for iron-cycling enhancement were S^2−^ and H_2_S; meanwhile, generated CH_3_C(O)OO^•^ and CH_3_C(O)O^•^ are significantly quenched by sulfur species and HO^•^ becomes the dominant ROS.

Differently from other heterogeneous systems, FeS is rapidly consumed (i.e., it works more as a reagent than as a catalyst), requiring repeated dosing every cycle (or its immobilization). This drawback can be overcome with the use of pyrite (FeS_2_); FeS_2_/PAA exhibited promising CEC abatement in neutral conditions, significantly faster than the analogous FeS_2_/H_2_O_2_ system [131]. The degradation of 10 μM tetracycline (degradation >99% in 30 min at pH 7.0 with 100 μM PAA and 100 mg L^−1^ pyrite) featured: *(i)* negligible contribution to CEC degradation by coexistent H_2_O_2_; *(ii)* CH_3_C(O)OO^•^ as the dominant ROS (as opposed to previously discussed work with FeS), and *(iii)* Fe(III) reduction agents (S_2_^2−^, S_5_^2−^, and S_8_^0^) eventually leading to SO_4_^2−^ as final product [131]. In this sense, iron-sulfur minerals containing other transition metals, such as chalcopyrite (CuFeS_2_), have also been recently applied towards PAA activation [132].

##### 3.3.2.2 Spinel ferrites

**Fig. 6:**
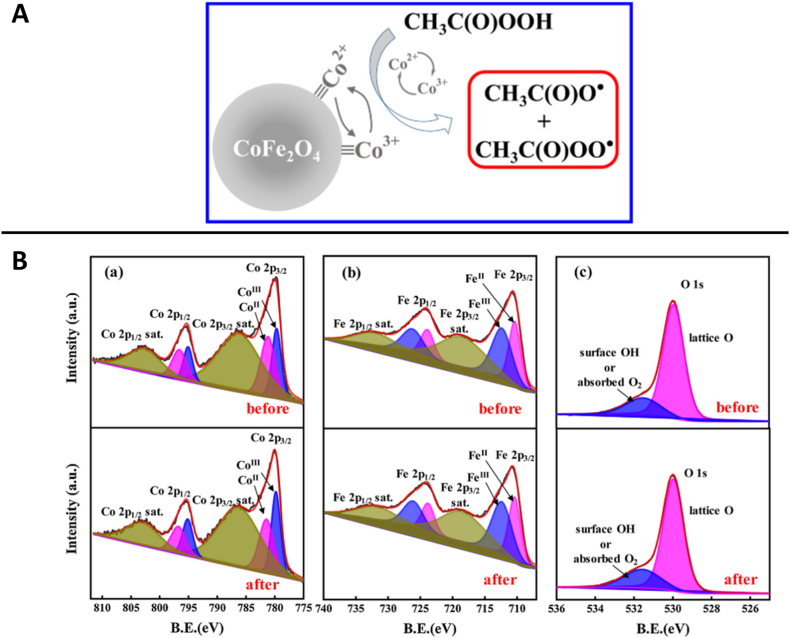
A) Mechanism of PAA activation by CoFe_2_O_4_ proposed by authors, and B) XPS spectra of CoFe_2_O_4_ before and after reaction with PAA. Figures reprinted with permission from Elsevier, J. Wang et al. (2021) [67].

Due to their hardness, corrosion resistance, strong magnetism (allowing magnetic separation, thereby facilitating reusability), and high catalytic efficiencies with other oxidants [133], spinel ferrites (MFe_2_O_4_) were also proposed as heterogeneous activators of PAA, CoFe_2_O_4_ being the most applied at the moment. One of the most detailed works by Wang et al. (2021) reported faster SMX degradation at neutral pH rather than in acidic or alkaline conditions, which was attributed to the change in surface charges of the spinel, modifying the interactions with PAA or PAA^−^ (CoFe_2_O_4_ isoelectric point is approximately 5.5). CoFe_2_O_4_ also exhibited high material reusability, and PAA decomposition was predominantly catalysed by Co(III)/Co(II) sites (see Fig. 6A) [67]. Oxidation states of CoFe_2_O_4_ measured by XPS were stated as mixed, due to the presence of Fe(II), Fe(III), Co(II), and Co(III). The predominant role of Co was evidenced by: *(i)* the scarce formation of HO^•^ when compared with CH_3_C(O)OO^•^ and CH_3_C(O)O^•^ (confirmed by selective scavenger experiments and electron paramagnetic resonance measurements), *(ii)* the lack of SMX degradation at neutral pH when using CoFe_2_O_4_/H_2_O_2_ (Co(II,III) does not activate H_2_O_2_ efficiently), *(iii)* a variation of 6% in the respective Co(II) and Co(III) fractions inside the material, before and after contact with PAA, which was negligible in the case of Fe(II) and Fe(III) (<1% change, see Fig. 6B). Noteworthy, it was suggested that Fe(III) gave higher electron density to the crystal planes, facilitating PAA adsorption and, therefore, also charge transfer. On the other hand, as a drawback, 0.25 μM Co_total_ was measured in solution after 30 min of CoFe_2_O_4_/PAA treatment [67], although contribution of homogeneous PAA activation by Co(II) was observed to be negligible. No data were reported in the cited work about Co leaching for longer treatments.

Other works suggested the support of CoFe_2_O_4_ on biochars, which are low-cost materials having large surface area (high adsorption capacity) and surface functional groups, usually derived from waste sources (e.g., sewage sludge or crop wastes). Dong et al. (2022) employed lignin-derived biochar and reported an enhancement of pollutant abatement compared to CoFe_2_O_4_ alone (although results do not show significant differences on pollutant abatement, as ca. 65% SMX degradation was obtained in both cases) [134]. Noteworthy, the biochar significantly reduced Co leaching by a factor of about 2 when compared with CoFe_2_O_4_ alone. The effect of pH or water constituents are in agreement with the results mentioned in the work by Wang et al., 2021 [67]: best pH conditions were the neutral ones, while negligible degradation was observed at pH 3 or 11; CH_3_C(O)OO^•^ and CH_3_C(O)O^•^ were the main reactive species (Co played a predominant role in PAA activation); the process has high reusability potential. On the other hand, it has been reported with another CoFe_2_O_4_@biochar that the main reactive species was ^1^O_2_ rather than ROS or CH_3_C(O)OO^•^/CH_3_C(O)O^•^ [136], a pathway supported by other works studying the Co/PAA systems [63,135]. Furthermore, differently from previous works where the optimal pH condition was usually 7, it was observed that the target CEC (tetracycline) exhibited faster degradations at acidic-neutral conditions, with the removal rates following the decreasing order pH 5 > pH 3 > pH 7 > pH 9 ≈ pH 11, which looks surprising to some extent. Noteworthy, the isoelectric point of the composite was determined to be 3.47 [136], explaining the differences with CoFe_2_O_4_ alone [133] or the work with lignin [134], and indicating an important parameter to tune in order to drive degradations at neutral conditions (more desirable in view of applications in real water matrices). The same work also reports the formation of Fe(IV) and Co(IV) [136], species not stated as the main responsible for CEC elimination in related works. However, it was not clear if these high-valent species might be formed from homogeneous contribution (leaching) rather than from the surface of the catalyst (which might also be possible as proved for other Fe-based materials [137]). Further investigation on role of high-valent iron on heterogeneous systems should be carried out.

When changing the spinel from CoFe_2_O_4_ to FeCo_2_O_4_, faster SMX removal at pH 7 and lower Co leaching were reported, indicating a greater activation of PAA compared to the case of the ferrite [138]. Similarly to CoFe_2_O_4_, FeCo_2_O_4_ exhibited analogous activation of PAA, decomposition only catalysed by Co(II)/Co(III), and organic radicals as the predominant reactive species. The role of iron, once again, was stated as facilitating the adsorption and charge transfer of PAA. Interestingly, XPS measurements indicated that iron on FeCo_2_O_4_ had an oxidation state of 3+, with negligible Fe^2+^ (differently from other spinels); therefore, cobalt exhibited a mixed oxidation state of +5/2 that allows for joint existence of Co(II) and Co(III). Authors employed a clever method to understand whether Fe(III) presence was beneficial, or not. SMX degradation by FeCo_2_O_4_/PAA was compared with Co_3_O_4_/PAA, and it was observed that with the latter the degradations were 5 times slower than with the spinel. Therefore, PAA adsorption capacity and surface electron transfer are detrimental parameters that can be significantly improved by the incorporation of iron in Co-oxides (confirmed by density functional calculations and cyclic voltammetry measurements). Electron-transfer ability (and higher amount of Co) was responsible for the higher efficiency of FeCo_2_O_4_ (80% SMX degradation in 10 min) compared to CoFe_2_O_4_ (40% SMX degradation in 10 min).

As cobalt-free alternatives, Yu et al. (2022) reported that CuFe_2_O_4_ gave considerable Rhodamine B decolorisation, with the highest efficiencies obtained in neutral conditions (pH 6 and 8, with >90% absorbance reduction in 1 h). Although not applied in environmental remediation studies yet, an interesting ferrite alternative is MgFe_2_O_4_, recently applied to oxidize lignin into value-added aromatic and dicarboxylic acid compounds under mild conditions, exhibiting good recyclability and environmental compatibility [140].

Surprisingly there is a lack of studies employing iron perovskites (MFeO_3_) or magnetite (inverse spinel), although they were widely employed as catalysts for H_2_O_2_-based AOPs [141,142]. Only one study has been recently published employing Fe_3_O_4_/PAA to degrade SMT, obtaining slow degradation (70% in 1 h at pH 3.0) that was enhanced by boron (>90% in 20 min), in line with the works revised in section 3.2.2.2, and good reusability for at least 5 cycles [66].

##### 3.3.2.3 Other alternatives

Other iron-containing heterogeneous catalysts employed for PAA activation are: *(i)* layered double hydroxides containing Co and Fe (CoFe-LDH), which showed similar benefits as bimetallic Fe/Co oxides and metals: high PAA activation by Co ions, enhanced Co(III) reduction by Fe(II), dominant role of organic radicals (RO^•^), and fast pollutant abatements at pH 7 [143]; *(ii)* iron anchored to graphitic carbon nitride (g-C_3_N_4_) [144]; *(iii)* iron-biochar materials (obtained by pyrolysis of vegetable wastes with iron salts) [145] with an operative role of the activated carbon phase that also catalyses PAA decomposition [29]; iv) ceramic membranes containing iron oxides, to minimize fouling in filtration systems by only adding PAA [146], and v) Fe-zeolites [147].

### 3.4 Effect of water constituents on Fe-PAA processes

In comparison to HO^•^ or SO_4_^•−^, CH_3_C(O)OO^•^ and CH_3_C(O)O^•^ have significantly lower reactivity against anions [23]. As a consequence, the performances of AOPs employing PAA are less sensitive to inorganic scavenging when compared to other oxidants.

Regarding carbonates (major and common AOPs interference), faster CEC removals were reported with UV/PAA in presence of HCO_3_^−^ due to parallel formation of CH_3_C(O)OO^•^ by reaction of CO_3_^•−^ with PAA [25]. Noteworthy, works studying PAA activation by transition metals (Fe, Cu, and Co, either as ions or metals) usually report considerable decreasing performances of the respective PAA-AOP processes in presence of (bi)carbonates (>1 mM) due to the formation of stable metal-CO_3_ complexes which hinder the catalytic cycle [67,122,148]. For Fe/PAA systems, HCO_3_^−^ is reported as a major interference due to the formation of Fenton-inactive complexes that hinder the activation of PAA, as opposed (or in addition) to the consensus of HO^•^ scavenging during the classical Fenton process.

In Table 3, stability constants of Fe(II,III) with the most frequent anions (carbonates, phosphates, chloride, sulfate, and DOM) are summarised. CO_3_^2−^ are the anions which can form the most stable complexes with iron: logβ(Fe(OH)(CO_3_)_(aq)_) = 7.7; logβ(Fe(CO_3_)_3_^3−^) = 22; Fe(HCO_3_)^2+^ is stated as unstable [149]. Although these complexes can keep iron dissolved at neutral pH values, they show to be Fenton-inactive and prevent the regeneration of Fe(II), which would otherwise close the catalytic loop. Furthermore, formation of FeCO_3_ (logKps = 10) represents an additional route of iron precipitation [87,122].

**Table 3:** Reported stability constants and solubility constants of iron complexes with major water constituents. Reported values are at T = 25 °C and ionic strength 0.1 mol kg^−1^.

Ligand	Reaction	logβ	References
Chloride	Fe^2+^ + Cl^−^ → FeCl^+^	0.5	[168,169]
	Fe^3+^ + Cl^−^ → FeCl^2+^	0.8	
	Fe^3+^ + 2 Cl^-^ → FeCl_2_^+^	1.0	
Phosphate	Fe^3+^ + H_2_PO_4_^−^ → FeH_2_PO_4_^2+^	3.5	[170]
	Fe^3+^ + HPO_4_^2−^ → FeHPO_4_^+^	8	
Sulfate	Fe^3+^ + SO_4_^2−^ → FeSO_4_^+^	2.3	[149,168]
	Fe^3+^ + 2 SO_4_^2−^ → Fe(SO_4_)_2_^-^	3.2	
Carbonate	Fe^3+^ + 3 CO_3_^2−^ → Fe(CO_3_)_3_^3-^	22	[149]
	Fe(OH)^2+^ + CO_3_^2−^ → Fe(OH)(CO_3_)_(aq)_	7.7	
	Fe^2+^ + CO_3_^2−^ → FeCO_3(s)_	10 (-logKsp)	
Fulvic acid	Fe^2+/3+^ + fulvic acid → Fe-fulvic acid	6	[153]
Humic acid	Fe^2+/3+^ + humic acid → Fe-humic acid	10	

Phosphates might also scavenge ROS (quenching kinetic rate constants with HO^•^ being k = (0.1–1) × 10^5^ M^−1^ s^−1^ [53]), an issue not to be neglected when employing phosphate buffers to stabilize pH. Similarly to carbonates, phosphates have also been reported as strong interferents during Fe/H_2_O_2_ and Fe/PAA processes, due to the formation of the inactive coordination complexes FeH_2_PO_4_^2+^ and FeHPO_4_^+^ (logβ = 3.5 and 8, respectively) [26,79,150]. In this regard, it is interesting to comment the work by Kim et al. (2022) who employed PICA/Fe(III)/PAA at pH 7 [84], mentioned in section 3.2.2.1. Whereas HCO_3_^−^ decreased to some degree pollutant abatement by the AOP, the H_2_PO_4_^−^/HPO_4_^2−^ ions completely hindered the process. It is important to highlight that the anions were 80 times more concentrated than the organic ligand, PICA, thus Fe(III)-PICA was a minor species. The difference between the influence of carbonates and phosphates could be related to the fact that, although CO_3_^2−^ can generate more stable complexes with Fe(III) than HPO_4_^2−^, the latter is in higher concentration at pH 7 (pKa(H_2_PO_4_^−^/HPO_4_^2−^) = 7.2 vs. pKa(HCO_3_^−^/CO_3_^2−^) = 10.3).

At high Cl^−^ concentrations (>0.1 M), the associated HO^•^ quenching becomes also relevant (k = 1 × 10^3^ M^−1^ s^−1^ at pH 7 [47]), as well as iron complexation. FeCl^+^ formation does not affect the Fenton kinetic rates, but FeCl^2+^ or FeCl_2_^+^ (logβFe-Cl ≤ 1) are Fenton-like inactive (although they are photoactive) and decrease the reaction effectiveness [151,152]. On the other hand, formed chlorine radicals (Cl^•^ and Cl_2_^•−^) can also lead to the formation of halogenated DBP, a matter of concern due to the potentially carcinogenic nature and high toxicity of these compounds [47].

Sulfate, which has negligible reaction kinetics with HO^•^ and barely interacts with iron (logβ(FeSO_4_^+^) = 2.3) usually does not affect the Fe/PAA processes. NO_2_^−^ (powerful HO^•^ scavenger, k = 1 × 10^10^ M^−1^ s^−1^) and NO_3_^−^ (not a ROS scavenger) could photo-generate reactive nitrogen species (RNS) such as NO^•^, NO_2_^•^, and ONOO^•^ [153]. RNS can react with CECs to promote alternative degradation pathways that might, for instance, compensate for HO^•^ scavenging by NO_2_^−^ [56].

The most significant CH_3_C(O)OO^•^/CH_3_C(O)O^•^ scavenger reported within PAA-AOP works is natural organic matter, and particularly the dissolved organic matter (DOM), which exhibits higher affinity to RO^•^ (k = 5.8 × 10^4^ L mg_C_^−1^ s^−1^) than to HO^•^ (k = 2.5 × 10^4^ L mg_C_^−1^ s^−1^) [25]. In addition, in the case of PAA-based photochemical processes, high DOM concentrations can also produce an inner filter effect and decrease the available photon flux inside the reactor (although a minor photosensitising contribution to CEC indirect degradation might occur) [153]. In this sense, DOM can also form stable complexes with transition metals (e.g., logK ≈ 10), decreasing their catalytic efficiency [93,154]. Noteworthy, it is well known that DOM (mostly humic acids) can enhance Fenton reaction at circumneutral pH, keeping iron dissolved and reducing Fe(III) into Fe(II) with their phenolic moieties, thereby forming Fenton-active complexes, differently from those with carbonates or phosphate [88,155].

### 3.5 Performance comparison between different Fe/PAA processes

Table 4 summarises the performance of different Fe/PAA systems for CEC abatement at neutral pH conditions, indicating a good trend of correlated results. With few exceptions, concentrations of 50–500 μM PAA and 100 μM Fe are usually applied to obtain fast degradation in ultra-pure water, with 10–20 μM initial pollutant concentration. For 15 μM of a model CEC, 60–75% degradation can be achieved in 2 h when employing 100 μM PAA and 100 μM Fe(II). If employing Fe(III), faster degradation can be obtained but only in presence of ferric ligands. The use of reducing agents, such as NH_2_OH, ABTS, B, or MoS_2_, does not seem to significantly improve the degradation process at pH 7. Noteworthy, in some of these works, only 1 μM of Fe(II) was employed; in these cases it is not possible to critically compare their efficiency against iron chelating agents like PICA or l-cysteine, where 50–500 μM Fe(III) was used. The combination FeO_4_^2−^/PAA shows outstanding results; noteworthy, based on the mechanistic aspects involved, the use of FeO_4_^2−^/PAA or FeO_4_^2−^/H_2_O_2_ at pH 7–8 is practically equal and the effect of PAA is only visible at pH 9, which limits its use to very few scenarios. The use of UV–visible light enhances homogeneous Fenton processes, which has not yet been explored for heterogeneous systems, except for one work combining ZVI with UV light. Interestingly, studied materials such as FeS_2_, Cu–Fe bimetal, or CuFe_2_O_4_ have shown promising results at neutral pH values. Studies of Fe/PAA on UWTP secondary/tertiary effluents are few, but they showed fast degradation without employing high concentrations of either PAA or iron.

**Table 4:** CEC degradation performance by different Fe/PAA systems obtained at neutral pH values (6.0 ≤ pH ≤ 8.0).

System	Model CEC	[CEC]_0_ (μM)	Oxidation (%)	Time (min)	pH_0_	[PAA]_0_ (μM)	Catalyst	Water matrix	Reference
Fe(II)	MB	15	60	120	7.1	100	100 μM of Fe(II)	Ultra-pure	[24]
	Naproxen		70						
	BPA		75						
	MB	16	25	30	7.0	52	50 μM of Fe(II)	Ultra-pure	[78]
	p-arsanilic acid	5	50	5	7.0	200	400 μM Fe(II)	Ultra-pure	[79]
			85	300	6.0			UWTP	
	As(III)	10	>99	<1	7.0	10	20 μM of Fe(II)	Ultra-pure	
ABTS/Fe(III)	Diclofenac	5	55	30	6.0	100	1 μM of Fe(III) + 25 μM of ABTS	Ultra-pure	[91]
NH_2_OH/Fe(II)	Naproxen	10	<1	10	7.0	300	1 μM of Fe(II) + 150 μM of NH_2_OH	Ultra-pure	[92]
	Diclofenac	5	25	10	6.0	100	1 μM of Fe(II) + 100 μM of NH_2_OH	Ultra-pure	[93]
PICA/Fe(III)	MB	15	90	10	7.0	500	50 μM of Fe(III) + 125 μM of PICA	Ultra-pure	[84]
	Naproxen		>99	8					
	SMX		>99	6					
L-cys/Fe(III)	SMX	10	90	60	7.0	500	500 μM of Fe(III) + 250 μM of l-cysteine	Ultra-pure	[87]
Gallic acid/Fe(III)	BPA	25	90	20	7.0	100	50 μM of Fe(III) + 100 μM of gallic acid	Ultra-pure	[88]
B/Fe(III)	BPA	22	>99	20	6.2	500	20 μM of Fe(III) + 0.1 g L^−1^ of B	Ultra-pure	[94]
MoS_2_/Fe(III)	SMX	10	10	15	7.0	300	100 μM of Fe(III) + 0.1 g L^−1^ of MoS_2_	Ultra-pure	[96]
Ferrate(VI)	Sulfadimethoxine	10	90	5	6.9	100	200 μM of FeO_4_^2-^	UWTP	[97]
	Trimethoprim		>99%						
	Carbamazepine		>99%						
	Carbamazepine	10	90	8.3	8.0	50	50 μM of FeO_4_^2-^	Ultra-pure	[98]
UV/Fe(II)	Triclosan	1	95	20	7.0	1000	10 μM of Fe(II)	Ultra-pure	[102]
	Acetominophen	132	70	30	7.0	3000	500 μM of Fe(II)	Ultra-pure	[26]
	Naproxen	21.7	5	15	7.0	100	2.5 μM of Fe(II)	Ultra-pure	[171]
VUV/Fe(II)	Carbamazepine	10	85	5	7.0	50	10 μM of Fe(II)	Ultra-pure	[104]
UV/Fe(III)	Rhodamine B	104	40	10	7.0	658	308 μM of Fe(III)	Ultra-pure	[103]
EC/Fe(II)	MB	62.5	50	60	7.0	5400	30 μM of Fe(II)	Ultra-pure	[110]
ZVI	Tetracycline	10	75	30	7.5	100	0.06 g L^−1^ of nZVI	Ultra-pure	[122]
UV/ZVI	Spiramycin	12	55	60	7.0	39	0.02 g L^−1^ of nZVI	Ultra-pure	[123]
S-ZVI	SMT	18	17.5	60	6.0	100	0.02 g L^−1^ of S-ZVI	Ultra-pure	[125]
	SMX	10	>99	15	7.0	200	0.1 g L^−1^ of S-ZVI	Ultra-pure	[126]
Cu^0^–Fe^0^	SMT	10	95	20	7.0	200	0.05 g L^−1^ of nZVI-Cu^0^	Ultra-pure	[128]
Co^0^–Fe^0^	SMX	20	>99	30	7.0	200	0.1 g L^−1^ of ZVI-Co^0^	Ultra-pure	[127]
FeS	SMX	10	95	10	7.0	100	0.025 g L^−1^ of FeS	Ultra-pure	[130]
FeS_2_	Tetracycline	10	>99	30	7.0	100	0.1 g L^−1^ of FeS_2_	Ultra-pure	[131]
CoFe_2_O_4_	SMX	10	75	30	7.0	100	0.1 g L^−1^ of CoFe_2_O_4_	Ultra-pure	[67]
	SMX	39	>99	20	7.0	550	0.1 g L^−1^ of CoFe_2_O_4_@biochar	Ultra-pure	[134]
	Tetracycline	22	70	60	7.0	600	1.0 g L^−1^ of CoFe_2_O_4_@biochar	Ultra-pure	[136]
FeCo_2_O_4_	SMX	10	90	20	7.0	100	0.1 g L^−1^ of FeCo_2_O_4_	Ultra-pure	[138]
CuFe_2_O_4_	Rhodamine B	42	90	60	7.0	526	0.1 g L^−1^ of CuFe_2_O_4_	Ultra-pure	[139]
Fe@g-C_3_N_4_	SMX	39	80	60	7.2	2000	0.4 g L^−1^ of Fe@g-C_3_N_4_	Ultra-pure	[144]

## 4 Environmental implications and future perspectives

Based on the available literature, we can conclude that PAA-Fenton processes seem to be more advantageous than the classic H_2_O_2_-Fenton ones, especially in complex water matrices (as far as pH and the presence of anions or DOM is concerned), which can be explained by faster reactions of Fe(II)/Fe(III) with PAA than with H_2_O_2_ and by the production of selective organic radicals (CH_3_C(O)OO^•^ and CH_3_C(O)O^•^). The cost of PAA is expected to decrease significantly in the next decade, in agreement with its rapidly increasing popularisation. However, the increase in total organic carbon content (residual PAA or HAc) is usually not considered but is a detrimental parameter that should be taken into account, together with formation of DBPs such as formaldehyde or trihalomethanes (the latter, mostly when dealing with highly saline wastewater). As a drawback, PAA is a liquid solution that, despite improvements in on-site production, is associated with higher costs linked to logistics and storage compared to solid reagents like percarbonate salts, PDS, PMS, or sulfite salts. The following issues are needed to be explored/borne in mind:

1. The properties of PAA solutions are highly pH-dependent due to *(i)* its “delicate” equilibrium with coexistent H_2_O_2_ and HAc (K_eq_ = 3.3) and *(ii)* its acid-base equilibrium (pKa_PAA_ = 8.2). These issues are relevant when choosing experimental conditions or when characterizing PAA solutions, because typical titrations (iodometry or ceriometry) use H_2_SO_4_ as catalyst, which might shift the PAA-H_2_O_2_ equilibrium and impair the measurement.
2. The current research on the use of performic acid or other peracids in AOPs is negligible. Due to similar chemical properties as PAA and higher cost-effectiveness towards wastewater disinfection, performic acid activation by transition metals or UV light deserves attention as a plausible novel AOP.
3. The iron chelating agents (which also act as iron cycling co-catalysts) picolinic acid and l-cysteine enhance the performances of Fe/H_2_O_2_ and/or Fe/PAA. However, their use is not as extended as that of NTA or EDDS.
4. Among iron compounds able to activate PAA, ferrate(VI) seems to be the most promising. Its mechanism (likely formation of complexes between PAA-Fe(IV)/Fe(V) species) needs further research. The addition of ferrate(VI) activators (i.e., sulfite, hydroxylamine, or graphene oxide) can extend even further the already effective FeO_4_^2−^/PAA treatment at circumneutral pH values. Real wastewater applications, pilot plant scale experiments, and life cycle assessments are certainly of interest.
5. Although electrical current generation will be greener (carbon-free) in the next few years, solar-photo-Fenton with PAA is an interesting option to replace UV lamps. Electro-Fenton and Sono-Fenton with PAA at circumneutral pH values are still fields to explore.
6. ZVI sulfidation significantly improves the corresponding Fenton performance at circumneutral pH values, due to Fe(III) reduction by sulfur-species. Analogous results were reported for FeS or FeS_2_.
7. The use of Cu–Fe as bimetal or as oxides is encouraged due to high synergy and plausible good efficiency for water treatment at neutral pH values. In contrast, employing Co (alone or combined with Fe) as a potentially promising catalyst for AOP is discouraged because it is a critical and toxic raw material.
8. Targets reported for Fe/PAA systems are mostly antibiotics or pharmaceuticals, with concentrations in the range of μM. Application of PAA activated by iron species must be extended to other pollutants (pesticides, nitroaromatics, halogenated compounds, *etc.*) and microorganisms, to fully cover its efficiency as water treatment technology. Moreover, testing Fe/PAA systems with real water matrices and CEC in the nM range must be carried out to evaluate the AOP performance in real scenarios.
9. There are no works analysing engineered systems in a systematic way for Fe/PAA processes: lack of life cycle assessment (LCA), study of Fe and/or PAA dosage (small and continuous/periodical additions vs. single ones), reactor designs (e.g., no studies employing well known CPC-reactors for photo-Fenton-PAA processes are reported so far, as well as raceways). Noteworthy, reactor-design results should not differ much from the already well-known Fe/H_2_O_2_ systems, explaining the lack of interest in this aspect.

## Data availability

Data will be made available on request.

## CRediT authorship contribution statement

**Iván Sciscenko:** Writing – review & editing, Writing – original draft, Visualization, Project administration, Investigation, Funding acquisition, Formal analysis, Data curation, Conceptualization. **Davide Vione:** Writing – review & editing, Validation, Supervision, Resources, Project administration, Funding acquisition, Conceptualization. **Marco Minella:** Writing – review & editing, Writing – original draft, Visualization, Validation, Supervision, Resources, Project administration, Funding acquisition, Conceptualization.

## Declaration of competing interest

The authors declare that they have no known competing financial interests or personal relationships that could have appeared to influence the work reported in this paper.
